# The impact of HIV prevention behavior patterns on infection risk among young men who have sex with men: a latent class analysis

**DOI:** 10.1186/s12889-026-27517-8

**Published:** 2026-04-30

**Authors:** Xiaoyan Zhang, Jingran Dong, Miaomiao Li, Xiaoping Zhan, Zhan Lin, Junfeng Zhang, Jie Yang, Maohe Yu, Jianyun Bai, Yi Liu, Changping Li, Zhuang Cui

**Affiliations:** 1 Department of Epidemiology and Health Statistics, School of Public Health, Tianjin Medical University, Tianjin, China; 2 Research and Development Department, Beijing BeMarcos Technology Co., Ltd., Beijing, China; 3 Tianjin Shenlan Public Health Counseling Service Center, Tianjin, China; 4 STD & AIDS Control and Prevention Section, Tianjin Center for Disease Control and Prevention, Tianjin, China; 5 Department of Epidemiology & Biostatistics, School of Public Health, Tianjin Medical University, Qixiangtai Road 22, Heping district, Tianjin, 300070 PR China

**Keywords:** Men who have sex with men, Latent class analysis, HIV prevention behaviors, Infection risk

## Abstract

**Objective:**

HIV infection rises rapidly in YMSM (young men who have sex with men). Despite extensive promotion of risk-reduction measures, existing research lacks categorical analysis of multidimensional prevention combinations, limiting precise interventions. This study uses latent class analysis (LCA) to identify prevention behavior patterns, analyze influencing factors, and evaluate association with HIV.

**Methods:**

From January 2021 to December 2024, 2,685 YMSM aged 15–24 in Tianjin were recruited through online and offline methods. 13 HIV-prevention indicators (one cognitive, twelve behavioral) were classified using LCA. Multinomial logistic regression analyzed associations between latent classes and demographics/intervention services factors, while differences in HIV infection across prevention behavior patterns were compared.

**Results:**

Four distinct HIV prevention classes emerged: “High-Risk Perception with Biomedical Reliance” (Class 1; 16.61%), “Low-Risk Perception with Condom Reliance” (Class 2; 16.72%), “Low-Risk Perception with Low Protection” (Class 3; 29.39%), and “High-Risk Perception with Partner & Barrier Strategy” (Class 4; 37.28%). Multinomial logistic regression showed that students were less likely to be classified into Class 1 (aOR = 0.56, 95%CI: 0.42–0.76) and Class 2 (aOR = 0.75, 95%CI: 0.57–0.97), and more likely to belong to Class 3. Online partner-seekers were less likely to fall into Class 2 (aOR = 0.58, 95%CI: 0.37–0.90) and Class 4 (aOR = 0.24, 95%CI: 0.17–0.36), and more prone to Class 3. For intervention services, those who received condom promotion or HIV counseling and testing were more likely to be categorized into Class 1 (aOR = 6.78, 95%CI: 4.20–10.95), Class 2 (aOR = 2.65, 95%CI: 1.86–3.77), and Class 4 (aOR = 10.64, 95%CI: 7.65–14.80), whereas peer education was negatively associated with Class 2 (aOR = 0.63, 95% CI: 0.44–0.91) and Class 4 (aOR = 0.34, 95% CI: 0.25–0.47). HIV analysis revealed Class 3 had the highest HIV positivity rate (6.60%, 95% CI: 5.08–8.77), followed by Class 1 (2.26%, 95% CI: 1.11–4.54) and Class 4 (2.20%, 95% CI: 1.20–3.32), with Class 2 showing the lowest rate (0.83%, 95% CI: 0.28–2.45).

**Conclusion:**

Among YMSM, HIV prevention behavior is highly heterogeneous. The highest-risk subgroup—“Low-Risk Perception with Low Protection”—should be prioritized, particularly among students and online partner-seekers. Peer education programs may require further optimization, while integrated prevention strategies should combine condom use, biomedical tools, and both online and offline delivery channels.

**Supplementary Information:**

The online version contains supplementary material available at 10.1186/s12889-026-27517-8.

## Introduction

HIV/AIDS remains a critical global public health challenge, particularly for men who have sex with men (MSM). This group continues to face a severe and disproportionate burden in the global epidemic, with significantly higher HIV infection rates and relative risk compared to the general population [1]. Among these, adolescents and young adults (aged 15–24) bear a disproportionate HIV/AIDS burden, representing one of the fastest-growing category of new infections globally, alongside rising mortality rates among youth due to AIDS-related complications [2]. In China, the proportion of homosexual transmission increased from 9.1% in 2009 to 23.3% in 2020 [3], with persistent upward trends. A study in Chengdu, China, found high new HIV infection rates among YMSM (young men who have sex with men) actively using geosocial networking (GSN) apps, especially mobile non-student MSM [4].

The increased risk of HIV infection among YMSM can be attributed to the fact that this group is at the intersection of sexual activity and social role transitions. Additionally, societal stigma and mental health pressures contribute to issues such as inconsistent condom use, insufficient risk awareness, and a disconnect between prevention knowledge and behavior [5–7]. Furthermore, the anonymity and convenience of online partner-seeking through internet-based platforms have further amplified high-risk sexual practices [4, 8]. This complex risk environment likely fosters diverse and often incongruent combinations of HIV prevention behaviors among YMSM. Therefore, precisely identifying these patterned prevention behaviors is critical to developing effective, tailored interventions that curb HIV transmission in this population.

In recent years, HIV prevention strategies have shifted from singular behavioral interventions to multimodal approaches. Traditional prevention efforts centered around condom promotion are gradually being integrated with biomedical interventions (such as pre-exposure prophylaxis (PrEP) and post-exposure prophylaxis (PEP)), regular testing, and partner notification [9]. Individual prevention choices are dynamically shaped by knowledge, risk perception, social support, and service accessibility [10–12]. For instance, some MSM may prioritize condoms while neglecting testing, whereas others recognize infection risks but rarely adopt biomedical interventions like PrEP or PEP. Such complex behavioral interactions challenge conventional univariate analyses in capturing the structural dynamics of prevention strategies, limiting precise targeting of high-risk subgroups.

Despite continued efforts to strengthen interventions among MSM through health education, community mobilization, and fixed-point testing, new infection rates among YMSM continues to rise [13]. This highlights limitations of existing “homogenized” intervention strategies in addressing challenges like knowledge-behavior disconnection, expansion of sexual networks via new social media, and barriers to PrEP/PEP accessibility.

Latent class analysis (LCA) offers a methodological breakthrough for analyzing such complex behavioral patterns. By identifying subgroups of individuals with similar behavioral characteristics, LCA enables differentiated intervention targeting [14]. Studies in Western MSM populations have applied LCA to identify risk classes such as “Unprotected-Anal-Intercourse”, “Partner-Seekers”, and “Multiple-Behaviors” demonstrating significant variations in sexually transmitted infection (STI) risks [15]. However, research applying LCA to young Chinese MSM remains scarce, hindering systematic understanding of HIV prevention behaviors and limiting the optimization of intervention resource allocation.

This study aimed to: 1) use LCA to identify latent classes of HIV prevention behaviors among YMSM in Tianjin, China, and reveal combined characteristics of multidimensional prevention strategies; 2) analyze the impact of demographic characteristics and intervention service exposure on class membership; 3) assess the epidemiological association between prevention behavioral classes and HIV infection outcomes. The findings will provide evidence to support the design of layered, targeted intervention strategies—tailored to each subgroup’s unique risk perception and protective behavior profiles through the identification of distinct prevention behavioral classes—and contribute to achieving UNAIDS’ “95-95-95” prevention target.

## Materials and methods

### Study design and participants

This study was based on an open cohort study conducted by Tianjin Center for Disease Control and Prevention (CDC) in collaboration with the Shenlan social organization. The cohort recruited MSM through combined online and offline strategies: online recruitment via social media platforms, and offline recruitment in counseling/testing services by providing free condoms/lubricants and encouraging peer referrals. Enrolled participants underwent 6-monthly follow-ups to collect sociodemographic, behavioral, and intervention service data, and receive HIV testing.

**Fig. 1:**
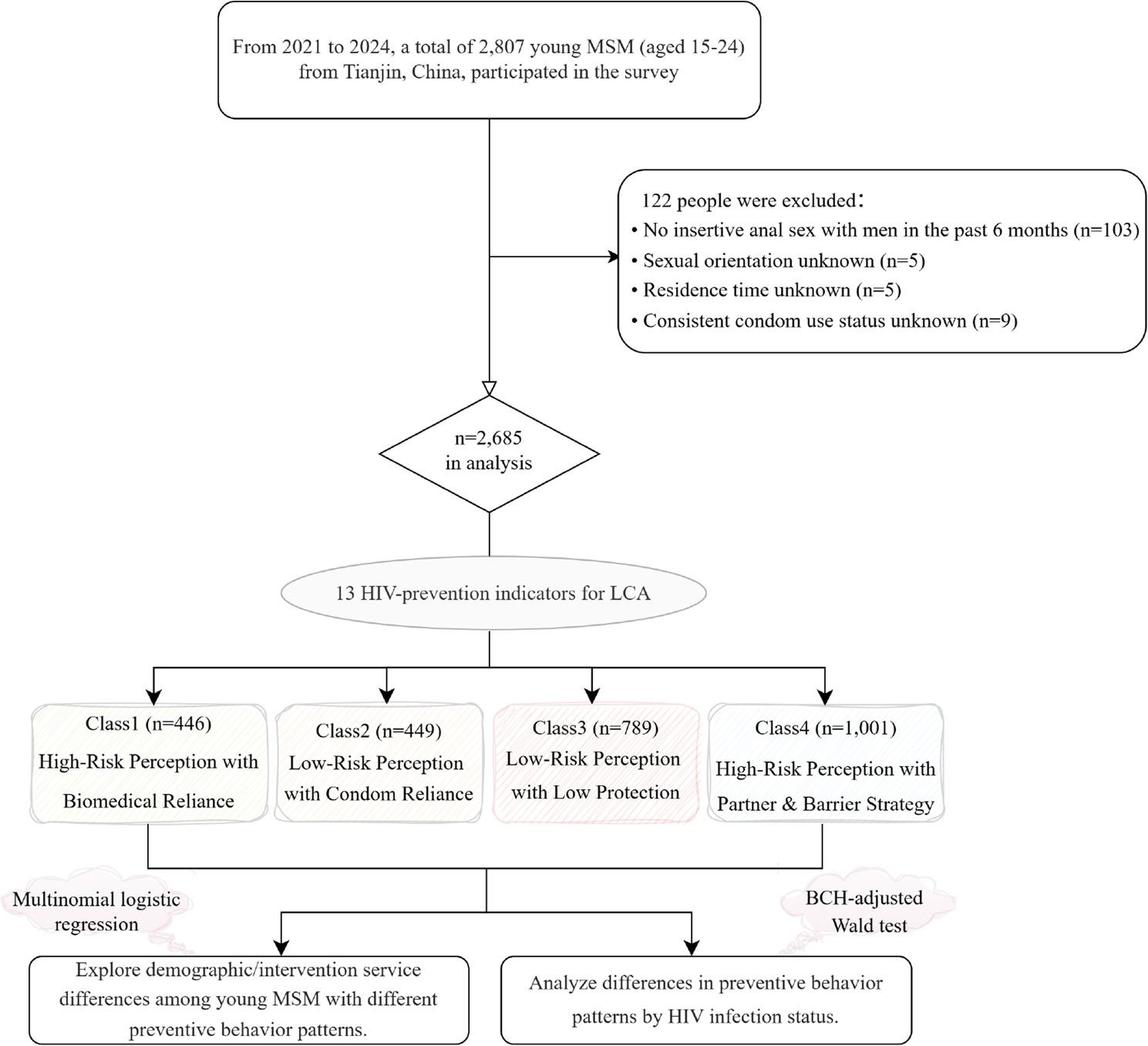
Flowchart of prevention behavior patterns for YMSM. 13 HIV-prevention indicators (LCA): 1 cognitive (self-perceived HIV risk), 12 behavioral (condom use during the most recent anal sex, consistent condom use, use of water-based lubricants, abstinence from recreational drugs, fixed sexual partners, regular on-site professional testing, partner testing, testing before anal sex, nucleic acid testing, knowledge of partner HIV status, use of PrEP, use of PEP). BCH-adjusted Wald test: Method to compare HIV differences across latent classes (corrects for latent class classification error)

To identify current HIV prevention behavior combinations and their cross-sectional association with HIV infection status, LCA was used on data from participants who completed at least one follow-up between January 2021 and December 2024. Inclusion criteria: 1) aged 15–24; 2) at least one insertive sexual act with a male in the past 6 months. Participants with missing data on sociodemographic or sexual behavior variables were excluded (all < 5% missingness). A total of 2,685 YMSM were included after fingerprint coding for identification and analysis of their last follow-up or HIV-positive confirmation records. The detailed exclusion and inclusion criteria for the participants are shown in Fig. 1.

### Study protocol

The study followed standardized procedures. All participants enrolled voluntarily without material rewards to ensure data authenticity. Investigators completed unified training on ethics, questionnaire logic verification, and biosafety operations, passing both theoretical and practical assessments. The questionnaire, designed by Tianjin CDC professionals, underwent rigorous validation (Cronbach’s α = 0.85, KMO = 0.8) through pretesting before formal use.

During data collection, investigators first recorded participants’ right index fingerprint to create an electronic file with a unique code for anonymization. Face-to-face questionnaires were administered using MSM-specific jargon to enhance trust and response accuracy. After questionnaire completion, venous blood was collected by qualified phlebotomists for HIV testing: initial screening with Beijing Wantai rapid test reagents, rechecking positive samples with Zhuhai Lizhu ELISA, confirming positive results by Singapore MP Bio Western Blot, and further quantifying uncertain/negative samples using Roche viral load testing. Non-reactive samples from initial and rechecking underwent pooled nucleic acid testing. Investigators reviewed questionnaires, assessed participant risks, and provided HIV/AIDS health education and behavior guidance. HIV test results were notified within one week, and positive participants were accompanied by volunteers for follow-up treatment and medication intervention. Participants who tested HIV-positive or had discrepant rapid tests underwent confirmatory testing using Western Blot and a Nucleic Acid Test. Those with confirmed positive results were guided by volunteer staff to access follow-up medical care and antiretroviral treatment.

### Survey questionnaire design

The survey questionnaire was developed specifically for this study and is provided as Supplementary Text 1. It included questions on demographic characteristics, cognitive and behavioral factors related to HIV prevention, HIV/AIDS knowledge, and receipt of HIV-related intervention services. Demographic characteristics included age, gender, race, marital status, student status, education, sexual orientation, residence time, and partner seeking source.

The cognitive factor was self-perception of high HIV risk (Yes/No). HIV prevention-related behaviors consisted of the following 12 risk-reduction practices: condom use during the most recent anal sex (Yes/No), consistent condom use (Yes/No), use of water-based lubricants (Yes/No), abstinence from recreational drugs (Yes/No), regular on-site professional testing (Yes/No), fixed sexual partners (Yes/No), partner testing (Yes/No), testing before anal sex (Yes/No), nucleic acid testing (Yes/No), knowledge of partner HIV status (Yes/No), use of PrEP (Yes/No), and use of PEP (Yes/No).

Participants who correctly answered ≥ 7 of 8 HIV/AIDS knowledge questions were classified as having high knowledge [16]. Receipt of HIV-related intervention services included condom promotion or HIV counseling testing and peer education.

### Ethics approval

The study was reviewed and approved by the Ethics Committee of Tianjin Medical University (approve number: TMUhMEC2021010). All participants signed an informed consent form before the survey started.

### Statistical analyses

Data entry was performed using EpiData 3.1 software, data organization and description were carried out using R 4.4.1, and LCA was conducted using SAS 9.4 software with PROC LCA (version 1.3.2). The LCA incorporated one cognitive indicator and twelve behavioral prevention indicators to identify latent prevention classes. The analysis began with a one-class model, and the number of classes was progressively increased to six to determine the best classification.

Model fit for the LCA was evaluated using the likelihood ratio test statistic (G²), the log-likelihood (LL), and information criteria. The information criteria included the Akaike information criterion (AIC), Bayesian information criterion (BIC), adjusted Bayesian information criterion (ABIC), and consistent Akaike information criterion (CAIC). Classification accuracy was measured using entropy, which ranges from 0 to 1, with values closer to 1 indicating higher classification precision [17, 18]. We also used the Lo–Mendell–Rubin likelihood ratio test (LMR-LRT) and the bootstrap likelihood ratio test (BLRT) to compare the k-class model with the k − 1 class model; a significant *P*-value (*P* < 0.05) indicates that the k-class model fits the data better than the k − 1 class model. Because no single index alone determines the optimal number of classes, the final model selection was guided by a balance among statistical fit, model parsimony, and interpretability of the latent classes.

After identifying the optimal latent class model, we examined factors associated with class membership using a three-step approach as described by Vermunt and Magidson [19, 20]. In this approach, the latent class model was first estimated without covariates. Individual posterior class-membership probabilities were then used to construct classification-error–adjusted weights. These weights were incorporated into a multinomial logistic regression model to assess associations between selected covariates and latent class membership, while accounting for classification uncertainty.

Potential covariates included demographic characteristics, behavioral factors, and HIV-related intervention services. Variables were first examined in univariate analyses, and those with *P* < 0.20 were considered candidates for multivariable modeling [21]. These candidate variables were then entered into a multivariable multinomial logistic regression model, and a stepwise selection procedure was applied to obtain a parsimonious final model, with *P* < 0.05 used as the criterion for variable retention. Results are reported as adjusted odds ratios (aOR) and 95% confidence intervals (CI). This analysis was implemented using the LCA Covariates 3-Step SAS macro (version 1.0) [22].

To evaluate differences in HIV infection across latent classes, we applied the Bolck–Croon–Hagenaars (BCH) method [23]. This approach accounts for classification error by applying BCH-adjusted weights to estimate class-specific outcome probabilities and to compare HIV infection across latent classes. Weighted estimates of HIV positivity with corresponding 95% CIs were obtained for each class, and overall differences between classes were assessed using BCH-adjusted Wald chi-square tests. These analyses were performed using the LCA_Distal_BCH SAS macro (version 1.1) [24].

## Results

### Study population

A total of 2,685 eligible YMSM were included, with a median age of 22.82 years (IQR: 21.17–24.06). The majority were Han nationality (*n* = 2,596, 96.69%), cisgender male (*n* = 2,680, 99.81%), single (*n* = 2,632, 98.03%), and 40.63% (*n* = 1,091) were students. Higher education was predominant (*n* = 2,087, 77.73%), with 94.64% (*n* = 2,541) self-identifying as gay. Most (*n* = 2,406, 89.61%) were long-term residents of Tianjin (> 6 months). A high proportion of participants sought sexual partners through online platforms (*n* = 2,326, 86.63%).

Regarding HIV prevention-related cognitive and behavioral factors: 32.10% (*n* = 862) reported high self-perceived HIV risk. For prevention behaviors, 47.00% (*n* = 1,262) used condoms during the most recent anal sex, and only 27.45% (*n* = 737) maintained consistent condom use; 40.78% (*n* = 1,095) used water-based lubricants. A minority abstained from recreational drugs (*n* = 457, 17.02%), and 22.16% (*n* = 595) had fixed sexual partners. In terms of testing behaviors: 25.81% (*n* = 693) underwent regular on-site professional testing, 11.51% (*n* = 309) reported partner HIV testing, 15.01% (*n* = 403) tested for HIV before anal sex, and 15.94% (*n* = 428) received nucleic acid testing. Additionally, 22.01% (*n* = 591) were aware of their partner’s HIV status. The utilization rates of pre-exposure prophylaxis (PrEP) and post-exposure prophylaxis (PEP) were 4.21% (*n* = 113) and 7.90% (*n* = 212), respectively.

HIV/AIDS knowledge awareness was high (*n* = 2,516, 93.71%). Approximately 71.28% (*n* = 1,914) had received condom promotion or HIV counseling testing services, and 46.26% (*n* = 1,242) reported participating in peer education. The overall HIV positivity rate of the sample was 3.17% (*n* = 85) (Table 1).

**Table 1:** Participant characteristic

**Characteristic**	**Overall **(*n* = 2,685)
Age (years)	22.82 (21.17–24.06)
Race, n (%)	
Han nationality	2,596 (96.69)
Ethnic minorities	89 (3.31)
Gender, n (%)	
Man	2,680 (99.81)
Transgender	5 (0.19)
Marital status, n (%)	
Single	2,632 (98.03)
Non-single	53 (1.97)
Student, n (%)	
Yes	1,091 (40.63)
No	1,594 (59.37)
Education, n (%)	
Postgraduate and above	214 (7.97)
Higher education	2,087 (77.73)
Secondary school	294 (10.95)
Primary school and below	90 (3.35)
Sexual orientation, n (%)	
Gay	2,541 (94.64)
Bisexual/Heterosexual	144 (5.36)
Residence time, n (%)	
>6 months	2,406 (89.61)
≤6 months	279 (10.39)
Partner seeking source, n (%)	
Online	2,326 (86.63)
Offline	359 (13.37)
Self-perceived HIV risk, n (%)	
Yes	862 (32.10)
No	1,823 (67.90)
Condom use during the most recent anal sex, n (%)	
Yes	1,262 (47.00)
No	1,423 (53.00)
Consistent condom use, n (%)	
Yes	737 (27.45)
No	1,948 (72.55)
Use of water-based lubricants, n (%)	
Yes	1,095 (40.78)
No	1,590 (59.22)
Abstinence from recreational drugs, n (%)	
Yes	457 (17.02)
No	2,228 (82.98)
Fixed sexual partners, n (%)	
Yes	595 (22.16)
No	2,090 (77.84)
Regular on-site professional testing, n (%)	
Yes	693 (25.81)
No	1,992 (74.19)
Partner testing, n (%)	
Yes	309 (11.51)
No	2,376 (88.49)
Testing before anal sex, n (%)	
Yes	403 (15.01)
No	2,282 (84.99)
Nucleic acid testing, n (%)	
Yes	428 (15.94)
No	2,257 (84.06)
Knowledge of partner HIV status, n (%)	
Yes	591 (22.01)
No	2,094 (77.99)
Use of PrEP, n (%)	
Yes	113 (4.21)
No	2,572 (95.79)
Use of PEP, n (%)	
Yes	212 (7.90)
No	2,473 (92.10)
HIV/AIDS awareness, n (%)	
Good	2,516 (93.71)
Poor	169 (6.29)
Condom promotion or HIV counseling testing, n (%)	
Yes	1,914 (71.28)
No	771 (28.72)
Peer education, n (%)	
Yes	1,242 (46.26)
No	1,443 (53.74)

Non-normal quantitative variables were presented as median (inter-quartile range), and qualitative variables were presented as the number of cases (component proportion)

### Model selection and class descriptions

Table 2 presents fit indices for one‑ to six‑class models. As the number of classes increased, G², AIC, BIC, CAIC, and ABIC progressively decreased, indicating improved fit. LMR-LRT and BLRT both showed significant improvements (all *P* < 0.05) up to six classes. Beyond statistical criteria, model selection also considered parsimony and interpretability. The three-class model failed to adequately distinguish key prevention behaviors, whereas the five- and six-class models produced conceptually redundant classes that fragmented existing patterns into minor variations without meaningful public health relevance. In contrast, the four-class solution yielded well-differentiated and theoretically coherent prevention behavior patterns with clear practical implications. Therefore, based on statistical fit, model parsimony, and substantive interpretability, the four-class solution was selected as the final model.

**Table 2:** Model fit indices for latent class analysis of HIV prevention behaviors

*N* classes	LOG_LIKELIHOOD	G_SQUARED	AIC	BIC	CAIC	ABIC	*P*_LMR−LRT_	*P*_BLRT_	ENTROPY
1	-17,010.21	8,784.995	8,810.995	8,887.636	8,900.636	8,846.331	-	-	-
2	-15,182.23	5,129.028	5,183.028	5,342.205	5,369.205	5,256.418	< 0.001	< 0.001	0.88
3	-14,808.27	4,381.118	4,463.118	4,704.831	4,745.831	4,574.561	< 0.001	< 0.001	0.81
4	-14,591.51	3,947.599	4,057.599	4,381.848	4,436.848	4,207.096	< 0.001	< 0.001	0.83
5	-14,408.76	3,582.093	3,720.093	4,126.878	4,195.878	3,907.644	< 0.001	< 0.001	0.83
6	-14,239.28	3,243.136	3,409.136	3,898.457	3,981.457	3,634.741	0.023	< 0.001	0.82

*Abbreviations*: *G*^2^ Likelihood ratio test statistic, *AIC* Akaike information criterion, *BIC* Bayesian information criterion, *CAIC* Consistent Akaike information criterion, *ABIC* Adjusted Bayesian information criterion, *LMR-LRT* Lo–Mendell–Rubin likelihood ratio test, *BLRT* Bootstrap likelihood ratio test

**Fig. 2:**
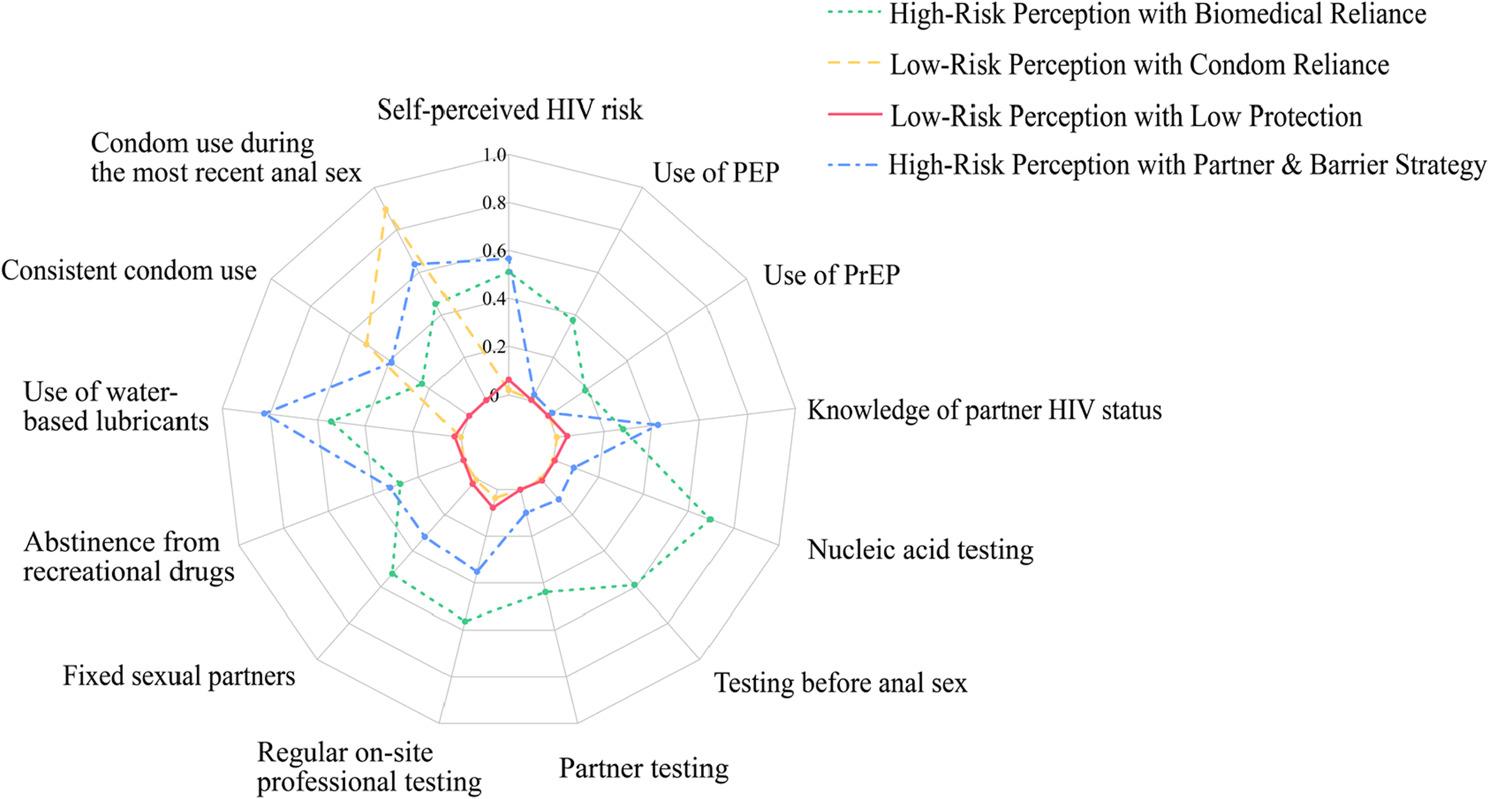
Conditional probability distributions of four latent classes. Item-response probabilities are the probabilities of participants endorsing different HIV prevention behaviors based on their latent class membership. For example, among those in Class 2 (Low-Risk Perception with Condom Reliance), the probability of using a condom during the most recent anal sex was 0.90

Figure 2 shows the results of the 4-class solution, and the specific prevalence rates of each prevention behavior indicator are provided in Supplementary Table 1. Class 1 is characterized by high-risk perception (self-perceived HIV risk probability of 51.03%) and biomedical prevention strategies, featuring the highest engagement in testing behaviors: nucleic acid testing (69.67%), pre-sex HIV testing (58.98%), regular professional testing (56.24%), and partner testing (43.90%). This group also demonstrates a high post-exposure prophylaxis (PEP) utilization rate (37.49%) and stable partnership (52.74%). Although the pre-exposure prophylaxis (PrEP) adoption rate remains low (18.65%), these ratios are significantly higher than those in other categories. Therefore, this category is named the “High-Risk Perception with Biomedical Reliance” accounting for 16.61% of the sample (*n* = 446).

Class 2, classified as “Low-Risk Perception with Condom Reliance”, which makes up 16.72% (*n* = 449) of the sample. They have a very low risk perception (2.15%), with almost universal condom use in the most recent anal sex (89.69%) and a consistent condom use rate of 51.74%. However, they hardly adopt other preventive measures: the probabilities of nucleic acid testing (0.03%), PrEP/PEP use (0.47%-0.52%), and partner testing (0.007%) are almost zero; the lubricant use rate is the lowest (0.09%).

Class 3 represents 29.39% (*n* = 789) of the included sample and was characterized by “Low-Risk Perception with Low Protection”. This subgroup has extremely low engagement in all preventive measures: the lowest frequency of consistent condom use (0.05%), hardly any testing behaviors (nucleic acid testing 0.52%, pre-sex testing 0.90%), and almost non-existent biomedical preventive measures (PrEP 0.002%, PEP 0.32%).

Class 4 is considered the “High-Risk Perception with Partner & Barrier Strategy”, accounted for 37.28% (*n* = 1,001) of the sample. This class showed the highest water-based lubricant use rate (82.35%) and a significant recent condom use rate (63.77%). Although this group reports the highest risk perception (56.54%), its engagement in biomedical interventions is limited (PrEP 2.12%, PEP 2.75%), and nucleic acid testing is only 9.00%.

### Factors associated with HIV prevention behavioral patterns

The distribution of HIV prevention behaviors among YMSM, based on demographic characteristics, HIV/AIDS knowledge levels, and the receipt of HIV-related intervention services, is presented in Supplementary Table 2. In univariate analyses, age, student status, sexual orientation, residence time, partner seeking source, HIV/AIDS knowledge, condom promotion or HIV counseling testing, and peer education were associated with latent class membership at *P* < 0.2 and were thus considered as candidate variables for the subsequent multivariable model.

Following stepwise selection, age, sexual orientation, residence time, and HIV/AIDS knowledge did not meet the criterion for retention (*P* < 0.05) and were therefore excluded from the final multivariable model. The variables retained in the final model were student status, partner seeking source, receipt of condom promotion or HIV counseling testing, and peer education.

**Fig. 3:**
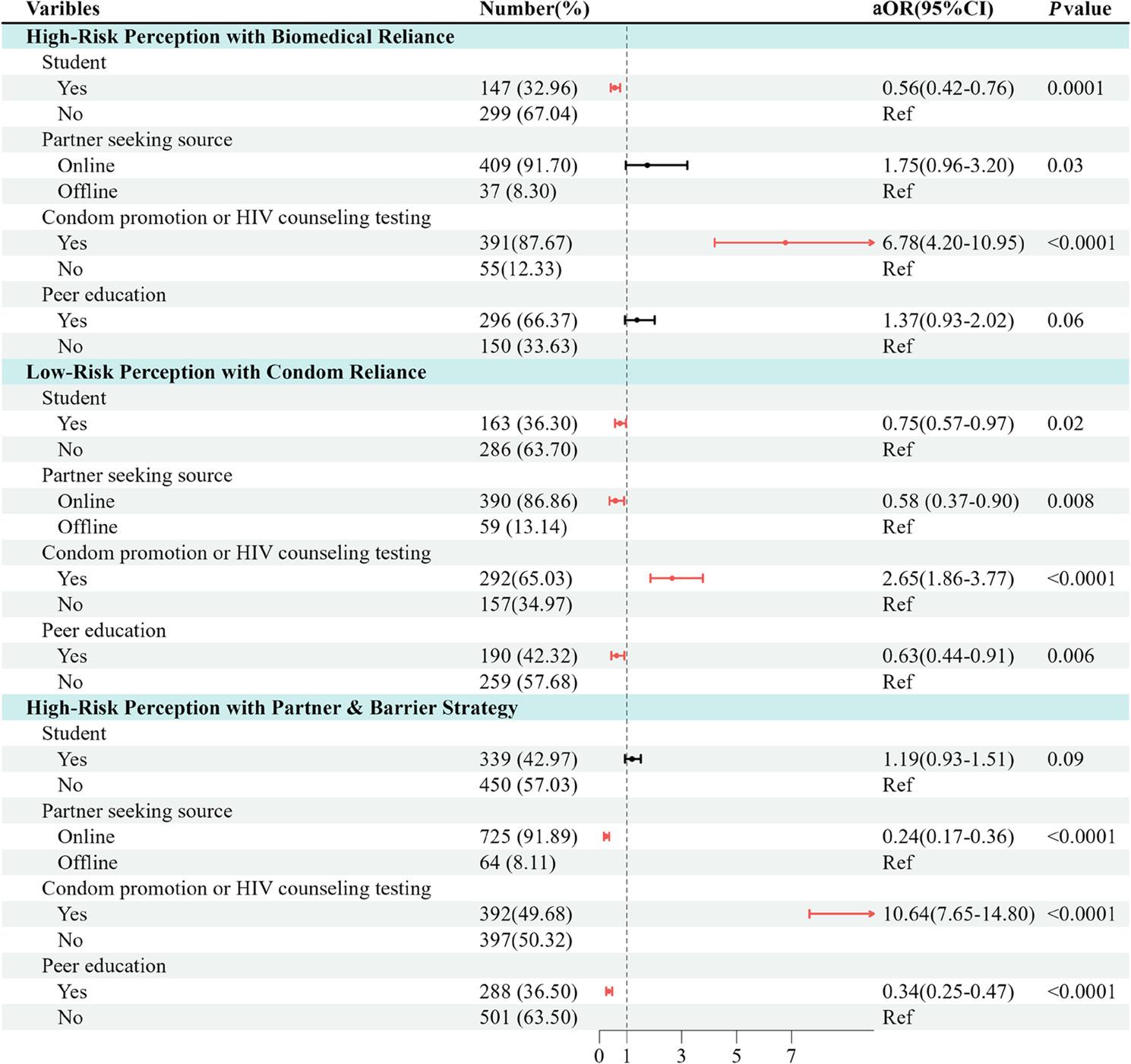
Factors associated with HIV prevention behaviors patterns using multinomial logistic regression. Class 3 (“Low-Risk Perception with Low Protection”) served as the reference group. aOR, adjusted odds ratio; 95% CI, 95% confidence interval. Red lines indicate statistically significant associations (95%CI of aOR excludes 1; *P* < 0.05), while black lines indicate non-statistically significant associations (95%CI of aOR includes 1; *P* ≥ 0.05). In rare cases, small sample sizes may result in *P* < 0.05 while the 95% CI still includes 1; these associations are shown as black lines

Using Class 3 (“Low-Risk Perception with Low Protection”) as the reference group, multinomial logistic regression revealed distinct associations between these covariates and latent class membership (Fig. 3). Students were less likely to belong to Class 1: “High-Risk Perception with Biomedical Reliance” (aOR = 0.56, 95% CI: 0.42–0.76, *P* = 0.0001) and Class 2: “Low-Risk Perception with Condom Reliance” (aOR = 0.75, 95% CI: 0.57–0.97, *P* = 0.02). Compared with offline partner-seeking, using online platforms was associated with significantly lower odds of belonging to Class 2 “Low-Risk Perception with Condom Reliance” (aOR = 0.58, 95% CI: 0.37–0.90, *P* = 0.008) and Class 4 “High-Risk Perception with Partner & Barrier Strategy” (aOR = 0.24, 95% CI: 0.17–0.36, *P* < 0.0001), but higher odds of membership in Class 1 “High-Risk Perception with Biomedical Reliance” (aOR = 1.75, 95% CI: 0.96–3.20, *P* = 0.03).

Among HIV-related intervention services, receipt of condom promotion or HIV counseling and testing was higher odds of membership in Class 1 “High-Risk Perception with Biomedical Reliance” (aOR = 6.78, 95% CI: 4.20-10.95, *P* < 0.0001), Class 2 “Low-Risk Perception with Condom Reliance” (aOR = 2.65, 95% CI: 1.86–3.77, *P* < 0.0001), and Class 4 “High-Risk Perception with Partner & Barrier Strategy” (aOR = 10.64, 95% CI: 7.65–14.80, *P* < 0.0001). Notably, peer education was associated with lower odds of membership in Class 2 “Low-Risk Perception with Condom Reliance” (aOR = 0.63, 95% CI: 0.44–0.91, *P* = 0.006) and Class 4 “High-Risk Perception with Partner & Barrier Strategy” (aOR = 0.34, 95% CI: 0.25–0.47, *P* < 0.0001). In contrast, no statistically significant association was observed with Class 1 “High-Risk Perception with Biomedical Reliance” (aOR = 1.37, 95% CI: 0.93–2.02, *P* = 0.06).

### Differential analysis of latent classes and HIV infection

**Fig. 4:**
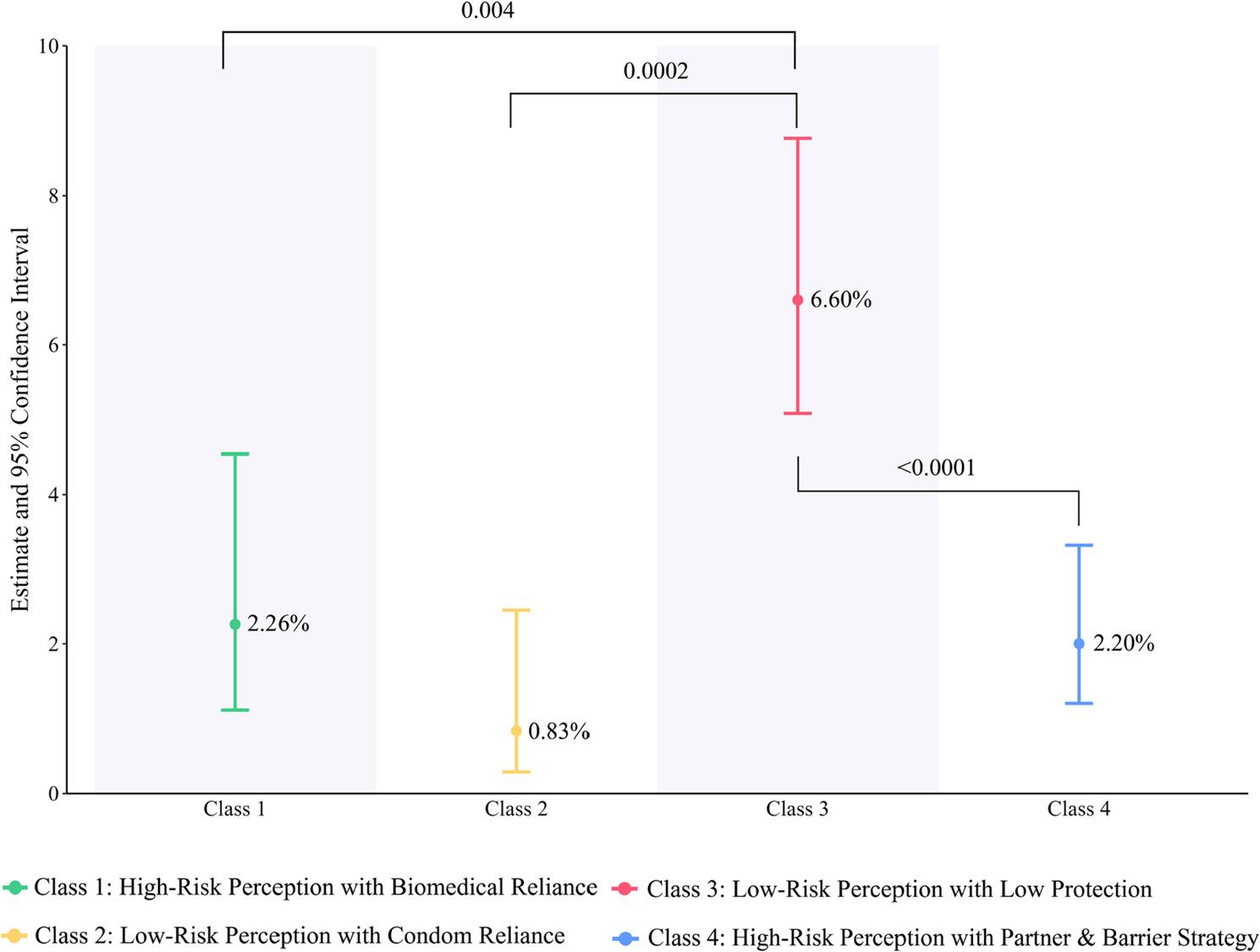
BCH-Estimated probabilities (95%CI) of HIV positivity rate across latent classes. Colored dots, HIV positivity rate estimates; error bars, 95% confidence intervals (95% CI). *P*-values (top), pairwise comparison results (BCH-adjusted Wald chi-square tests, Bonferroni-corrected α=0.008)

The BCH distal outcome analysis revealed significant differences in HIV positivity across latent prevention patterns (*P* < 0.0001). Class 3 “Low-Risk Perception with Low Protection” showed the highest HIV positivity (6.60%, 95% CI: 5.08–8.77). The estimated positivity in Class 3 was higher than that observed in Class 1 “High-Risk Perception with Biomedical Reliance” (2.26%, 95% CI: 1.11–4.54; *P* = 0.004), Class 2 “Low-Risk Perception with Condom Reliance” (0.83%, 95% CI: 0.28–2.45; *P* = 0.0002), and Class 4 “High-Risk Perception with Partner & Barrier Strategy” (2.00%, 95% CI: 1.20–3.32; *P* < 0.0001) (Fig. 4, Supplementary Table 3).

## Discussion

This study applied LCA to identify distinct patterns of HIV prevention behaviors among YMSM in Tianjin, China. We identified four distinct prevention behavioral patterns: “High-Risk Perception with Biomedical Reliance” (16.61%), “Low-Risk Perception with Condom Reliance” (16.72%), “Low-Risk Perception with Low Protection” (29.39%), and “High-Risk Perception with Partner & Barrier Strategy” (37.28%). The “Low-Risk Perception with Low Protection” class had the highest HIV positivity rate (6.60%), highlighting it as the highest-risk subgroup requiring urgent intervention. These findings underscore the significant heterogeneity in prevention practices within YMSM and demonstrate the utility of LCA in identifying subgroup-specific intervention needs beyond homogenized approaches.

Our analysis showed that students were less likely to belong to Class 1 “High-Risk Perception with Biomedical Reliance” and Class 2 “Low-Risk Perception with Condom Reliance”, which may suggest potential limitations of school-based sex and HIV prevention education, particularly in practical guidance on condom use and biomedical prevention. The collective living environment is also associated with increased concerns about peer visibility when engaging in prevention behaviors, such as frequent HIV testing, PrEP/PEP use, or obtaining condoms [25]. Compared with seeking partners offline (e.g., bars, bathhouses), online partner-seeking was more strongly associated with Class 3 “Low-Risk Perception with Low Protection,” possibly reflecting reduced perceived accountability and more casual prevention practices. In contrast, offline settings were associated with preventive behaviors through greater visibility of condom carrying and lubricant use, which may contribute to normative effects [26]. Notably, a non-significant trend indicated that online partner-seeking was also more likely to be associated with Class 1 “High-Risk Perception with Biomedical Reliance” (aOR = 1.75, 95% CI: 0.96–3.20), suggesting digital platforms may facilitate access to biomedical prevention information or services for some YMSM [27], a hypothesis that warrants further investigation in studies with larger sample sizes.

The associations between HIV intervention services and prevention behavioral classes varied across groups. Condom promotion or HIV counseling testing was positively associated with membership in the three classes exhibiting preventive behaviors (Classes 1, 2, and 4), suggesting that basic community services may provide protective tools and help reinforce comprehensive prevention awareness through regular contact [28]. In contrast, peer education showed heterogeneous associations with class membership: it was negatively associated with Class 2 “Low-Risk Perception with Condom Reliance” and Class 4 “High-Risk Perception with Partner & Barrier Strategy,” and not significantly associated with Class 1 “High-Risk Perception with Biomedical Reliance.” This suggests that the association between peer education and preventive behavior patterns may depend on recipients’ existing behavioral profiles [29, 30]. Oversimplified educational content has been associated with misunderstandings about condom use or PrEP, potentially influencing preventive choices and reducing overall effectiveness [31, 32].

A unique and critical finding was the exceptionally low HIV positivity rate (0.83%) in the “Low-Risk Perception with Condom Reliance” class, which supports the potential role of condoms as a highly effective, immediate, and low-cost barrier method. Conversely, the “High-Risk Perception with Biomedical Reliance” class, despite high testing rates, had a significantly higher HIV positivity rate (2.26%) than the condom-reliant group. This may indicate that reliance on biomedical strategies alone (without consistent condom use) may be associated with a lower level of protection. Prior research has shown that relying solely on PrEP requires an 80% coverage rate to eliminate transmission, while the combination of condoms and PrEP can achieve the goal ahead of schedule [33]. Therefore, promoting comprehensive prevention—a combination of consistent condom use, regular and partner testing, and access to PrEP/PEP where appropriate—may help establish a multi-layered HIV prevention system.

In addition, the identification of the “High-Risk Perception with Partner & Barrier Strategy” class further reflects the heterogeneity of prevention pathways among YMSM. Despite elevated self-perceived HIV risk, individuals in this class primarily relied on condoms and lubricants, with limited uptake of biomedical prevention and testing. This gap between risk awareness and biomedical strategy adoption may be associated with structural and psychosocial barriers, including stigma-related concerns, limited access to institution-based services, and economic constraints [34–36]. Previous studies suggest that misconceptions surrounding PrEP and PEP—such as viewing them as “licenses for promiscuity”—may reduce uptake and may be associated with greater reliance on self-managed barrier methods [37–40]. Avoidance of clinical settings due to stigma or psychological distress has also been documented among sexual minority populations, which may limit engagement with testing and biomedical prevention [41]. Additionally, the institutional nature of PrEP/PEP delivery, concerns about side effects, and long-term cost burdens may also be associated with lower uptake among YMSM [42]. Although this class did not exhibit the highest HIV positivity rate, its prevention profile may indicate opportunities to better integrate biomedical tools into existing protective behaviors, suggesting the need for context-sensitive strategies that reduce access barriers while respecting individuals’ preferred prevention approaches.

A key contribution of this study lies in the systematic examination of HIV risk perception alongside a comprehensive set of prevention behaviors among YMSM using latent class analysis. By identifying distinct combinations of traditional barrier methods, biomedical strategies, and insufficient protection, this study highlights the heterogeneity of prevention pathways within this population. These findings extend prior research that has largely focused on single preventive behaviors and provide a more nuanced framework for understanding how risk perception aligns with prevention choices among YMSM.

This study has certain limitations. First, data were collected from 2021 to 2024, during which the popularization of PrEP/PEP and self-testing may have potentially influenced behaviors. Although the latest survey data were used, associations between prevention behavioral patterns and HIV infection may still be affected. Future longitudinal methods, such as Latent Transition Analysis or Growth Mixture Model could be used to track class transitions over time and enable dynamic interventions. Second, the sample was limited to YMSM in Tianjin, and regional culture or service accessibility may limit generalizability. Finally, reliance on self-reported sexual behaviors and prevention measures may introduce information bias.

## Conclusion

This study provides a nuanced, subgroup-based perspective on HIV prevention among YMSM. To reduce new infections, public health programs should prioritize the high-risk “Low-Risk Perception with Low Protection” subgroup through targeted outreach, especially among students and online partner-seekers. Intervention strategies must be differentiated: condom promotion and testing access should be strengthened universally, while peer education programs require critical review and optimization to ensure they deliver accurate, comprehensive, and relevant messaging. Finally, health systems should promote integrated prevention packages that combine condoms, testing, and biomedical tools, adapted to the specific risk profile and context of each subgroup, thereby advancing progress toward the UNAIDS 95-95-95 targets. 

## Supplementary Material

Supplementary Material 1.

## Data Availability

The datasets used and analyzed during the current study are available from the corresponding author on reasonable request.
