# Supplementary material for: The impact of HIV prevention behavior patterns on infection risk among young men who have sex with men: a latent class analysis
Source: BMC Public Health. 2026 Apr 30;26:1916. doi: 10.1186/s12889-026-27517-8 (PMC13281564; doi:10.1186/s12889-026-27517-8)
Supplement: Supplementary file 1 — Supplementary Material 1. [file 12889_2026_27517_MOESM1_ESM.docx]

## SUPPLEMENTARY FILE

Table of contents:

|  |  | **Page** |
| --- | --- | --- |
| Supplementary Text 1 | Supplementary survey questionnaire. | 2-8 |
| Supplementary Table 1 | Item response and classification probabilities,4-class model | 9-10 |
| Supplementary Table 2 | Univariate analyses of associations between HIV prevention behavioral patterns, socio-demographics, and intervention services | 11-15 |
| Supplementary Table 3 | BCH-estimated probabilities (95%CI) of HIV positivity rate across latent class | 16 |

**Supplementary Text 1 Supplementary survey questionnaire**

**Part A: Demographic and Sociobehavioral Characteristics**

A1. Please provide your cellphone number: ____________________. By doing so, you voluntarily participate in this HIV test and survey. [Fill in the blank]

A2. What is your nickname? [Fill in the blank]

____________________

A3. Please enter your date of birth (YYYY-MM-DD) [Fill in the blank]

____________________

A4. Please confirm your gender. [Select one from]

(1) Male

(2) Female

(3) Transgender

A5. What is your nationality?

(1) Han

(2) Other (Please specify: ____________________)

A6. What is your current marital status? [Select one from]

(1) Unmarried

(2) Married

(3) Divorced/widowed

A7. Are you currently a student? [Select one from]

(1) Yes

(2) No

A8. What is your highest level of education? [Select one from]

(1) Primary school and below

(2) Secondary school

(3) High school

(4) College and above

A9. What is your sexual orientation? [Select one from]

(1) Homosexual (Gay)

(2) Bisexual

(3) Heterosexual

A10. How long have you resided in Tianjin? [Select one from]

(1) >6 months

(2) ≤6 months

A11. What is your primary channel for seeking sexual partners? [Select one from]

(1) Bar / Nightclub

(2) Tea House / Club

(3) Bathhouse

(4) Park / Public Toilet / Lawn

(5) Internet / Dating Apps

(6) Other venues (Please specify: ____________________)

**Part B: HIV/AIDS Knowledge Assessment**

*(Instructions: Please select the most accurate answer for each question.)*

B1. Is AIDS an incurable and serious infectious disease?

(1) Yes

(2) No

(3) Don't know

B2. Are men who have sex with men (MSM) one of the key populations most affected by HIV/AIDS in China currently?

(1) Yes

(2) No

(3) Don't know

B3. Can you tell if someone is infected with HIV by their appearance?

(1) Yes

(2) No

(3) Don't know

B4. Does being infected with other sexually transmitted diseases (STDs) increase the risk of HIV infection?

(1) Yes

(2) No

(3) Don't know

B5. Can consistent and correct condom use reduce the risk of acquiring or transmitting HIV?

(1) Yes

(2) No

(3) Don't know

B6. Does using new-type drugs (e.g., methamphetamine, ecstasy, ketamine) increase the risk of HIV infection?

(1) Yes

(2) No

(3) Don't know

B7. After engaging in high-risk behaviors (e.g., sharing needles for drug use, unprotected sex), should one actively seek HIV testing and counseling?

(1) Yes

(2) No

(3) Don't know

B8. Is intentional transmission of HIV punishable by law?

(1) Yes

(2) No

(3) Don't know

**Part C: Intervention Services Exposure**

*(Instructions: In the past year, have you received the following HIV prevention services?)*

C1. Condom promotion and distribution / HIV counseling and testing

(1) Yes

(2) No

C2. Peer education

(1) Yes

(2) No

**Part D: Sexual and Prevention Behaviors**

D1. Did you use a condom during your most recent anal intercourse (in the past 6 months)? [Select one from]

(1) Yes

(2) No

D2. How frequently did you use condoms during anal sex in the past 6 months? [Select one from]

(1) Never

(2) Sometimes

(3) Always

D3. Have you used risk reduction strategies other than using condoms? [Select one from]

(1) Yes

(2) No (If No, skip to End of Survey)

D4. Which of the following methods have you used? [Select all that apply]

(1) Self-perception of high HIV risk

(2) Knowledge of sexual partner's HIV test results

(3) Use of water-based lubricants

(4) Avoiding recreational drug use

(5) Regular sexual partnership

(6) Regular on-site HIV testing

(7) Ensuring partner undergoes HIV testing

(8) Taking an HIV test before sexual encounters

(9) HIV nucleic acid testing

(10) Using Pre-Exposure Prophylaxis (PrEP)

(11) Using Po st-Exposure Prophylaxis (PEP)

**Supplementary Table 1 Item response and classification probabilities,4-class model**

| **Item-Response Probabilities by Class** | **1** | **2** | **3** | **4** |
| --- | --- | --- | --- | --- |
| Total | 0.179089 | 0.171981 | 0.281381 | 0.367550 |
| Self-perceived HIV risk | 0.510320 | 0.021454 | 0.064472 | 0.565449 |
| Condom use during the most recent anal sex | 0.451337 | 0.896919 | 0.001888 | 0.637723 |
| Consistent condom use | 0.235443 | 0.517359 | 0.000452 | 0.389649 |
| Use of water-based lubricants | 0.542671 | 0.000905 | 0.027799 | 0.823505 |
| Abstinence from recreational drugs | 0.280157 | 0.000708 | 0.001370 | 0.325212 |
| Fixed sexual partners | 0.527437 | 0.005687 | 0.027344 | 0.322328 |
| Regular on-site professional testing | 0.562371 | 0.035796 | 0.077341 | 0.352247 |
| Partner testing | 0.438953 | 0.000065 | 0.000048 | 0.099144 |
| Testing before anal sex | 0.589825 | 0.000094 | 0.009042 | 0.113974 |
| Nucleic acid testing | 0.696715 | 0.000284 | 0.005241 | 0.090035 |
| Knowledge of partner HIV status | 0.280637 | 0.001937 | 0.045037 | 0.426769 |
| Use of PrEP | 0.186489 | 0.005190 | 0.000017 | 0.021184 |
| Use of PEP | 0.374915 | 0.004694 | 0.003173 | 0.027493 |

**Supplementary Table 2** **Univariate analyses of associations between HIV prevention behavioral patterns, socio-demographics, and intervention services**

| **Characteristic** | **Class1:**  **High-Risk Perception with Biomedical Reliance** (n = 446) | **Class2：**  **Low-Risk Perception with Condom Reliance** (n = 449) | **Class3:**  **Low-Risk Perception with Low Protection** (n = 789) | **Class4:**  **High-Risk Perception with Partner & Barrier Strategy** (n = 1,001) | ***P* value** |
| --- | --- | --- | --- | --- | --- |
| Age (years) | 22.75 (21.28–24.08) | 23.13 (21.41–24.24) | 22.77 (21.07–24.05) | 22.73 (21.14–23.98) | **0.065** |
| Gender, n(%) |  |  |  |  | 0.35 |
| Man | 446 (100.00) | 449 (100.00) | 788 (99.87) | 997 (99.60) |  |
| Transgender | 0 (0.00) | 0 (0.00) | 1 (0.13) | 4 (0.40) |  |
| Race, n(%) |  |  |  |  | 0.23 |
| Han nationality | 438 (98.21) | 434 (96.66) | 759 (96.20) | 965 (96.40) |  |
| Ethnic minorities | 8 (1.79) | 15 (3.34) | 30 (3.80) | 36 (3.60) |  |
| Student, n(%) |  |  |  |  | **<0.001** |
| Yes | 147 (32.96) | 163 (36.30) | 339 (42.97) | 442 (44.16) |  |
| No | 299 (67.04) | 286 (63.70) | 450 (57.03) | 559 (55.84) |  |
| Marital status, n(%) |  |  |  |  | 0.84 |
| Single | 436 (97.76) | 439 (97.77) | 773 (97.97) | 984 (98.30) |  |
| Non-single | 10 (2.24) | 10 (2.23) | 16 (2.03) | 17 (1.70) |  |
| Education, n(%) |  |  |  |  | 0.64 |
| Postgraduate and above | 32 (7.17) | 44 (9.80) | 61 (7.73) | 77 (7.69) |  |
| Higher education | 342 (76.68) | 347 (77.28) | 609 (77.19) | 789 (78.82) |  |
| Secondary school | 53 (11.88) | 48 (10.69) | 89 (11.28) | 104 (10.39) |  |
| Primary school and below | 19 (4.26) | 10 (2.23) | 30 (3.80) | 31 (3.10) |  |
| Sexual orientation, n(%) |  |  |  |  | **0.007** |
| Gay | 409 (91.70) | 422 (93.99) | 748 (94.80) | 962 (96.10) |  |
| Bisexual/Heterosexual | 37 (8.30) | 27 (6.01) | 41 (5.20) | 39 (3.90) |  |
| Residence time, n(%) |  |  |  |  | **0.004** |
| >6 months | 411 (92.15) | 411 (91.54) | 714 (90.49) | 870 (86.91) |  |
| ≤6 months | 35 (7.85) | 38 (8.46) | 75 (9.51) | 131 (13.09) |  |
| Partner seeking source, n(%) |  |  |  |  | **<0.001** |
| Offline | 37 (8.30) | 59 (13.14) | 64 (8.11) | 199 (19.88) |  |
| Online | 409 (91.70) | 390 (86.86) | 725 (91.89) | 802 (80.12) |  |
| HIV/AIDS awareness, n(%) |  |  |  |  | **<0.001** |
| Good | 425 (95.29) | 422 (93.99) | 715 (90.62) | 954 (95.30) |  |
| Poor | 21 (4.71) | 27 (6.01) | 74 (9.38) | 47 (4.70) |  |
| Condom promotion or HIV counseling testing, n(%) |  |  |  |  | **<0.001** |
| Yes | 391 (87.67) | 292 (65.03) | 392 (49.68) | 839 (83.82) |  |
| No | 55 (12.33) | 157 (34.97) | 397 (50.32) | 162 (16.18) |  |
| Peer education, n(%) |  |  |  |  | **<0.001** |
| Yes | 296 (66.37) | 190 (42.32) | 288 (36.50) | 468 (46.75) |  |
| No | 150 (33.63) | 259 (57.68) | 501 (63.50) | 533 (53.25) |  |

**Supplementary Table 3 BCH-estimated probabilities (95%CI) of HIV positivity rate across latent class**

| **Class** | **Probability % (95%*CI %*)** | **Pairwise Comparisons (*P*-value)** | | | |
| --- | --- | --- | --- | --- | --- |
|  |  | vs Class 1 | vs Class 2 | vs Class 3 | vs Class 4 |
| Class 1:  High-Risk Perception with Biomedical Reliance | 2.26 (1.11-4.54) | - | 0.13 | 0.004 | 0.80 |
| Class 2:  Low-Risk Perception with with Condom Reliance | 0.83 (0.28-2.45) | 0.13 | - | 0.0002 | 0.16 |
| Class 3:  Low-Risk Perception with Low Protection | 6.60 (5.08-8.77) | **0.004** | **0.0002** | - | <0.0001 |
| Class 4:  High-Risk Perception with Partner & Barrier Strategy | 2.00 (1.20-3.32) | 0.80 | 0.16 | **<0.0001** | - |

*P*-values were derived from BCH-adjusted Wald chi-square tests. Bonferroni correction was applied for multiple pairwise comparisons (adjusted α = 0.008).
